# Gradient Ethanol Precipitation of *Sanghuangporus vaninii* Polysaccharides: Physicochemical Properties and Inhibition of Tumor Cell Proliferation

**DOI:** 10.3390/foods15193474

**Published:** 2026-09-28

**Authors:** Xilei Guo, Jiayu Gu, Jiaxin Chu, Yuanqi Duan, Debin Wang, Hao Wu, Wei Zhou, Zhengyu Hu

**Affiliations:** 1 Key Laboratory of Natural Medicines of the Changbai Mountain, Ministry of Education, College of Pharmacy, Yanbian University, Yanji 133002, China; lxxx726@163.com (X.G.);; 2 Yanbian Special Industry Development Center, Yanji 133002, China; 3 Department of Pharmacy, Jilin Medical University, Jilin 132013, China

**Keywords:** *Sanghuangporus vaninii* polysaccharide, gradient ethanol precipitation, inhibition of tumor cell proliferation

## Abstract

Three polysaccharide fractions (SH-40, SH-60, and SH-80) were sequentially separated from *Sanghuangporus vaninii* fruiting bodies through graded ethanol precipitation to investigate the influence of molecular weight on their structure, physicochemical properties, and inhibition of tumor cell proliferation. The results revealed that the molecular weights of these three fractions were approximately 5.90 × 10^5^, 4.82 × 10^5^, and 4.13 × 10^5^ Da, respectively; they shared similar functional group profiles, monosaccharide compositions and glycosidic bonds. As the molecular weight decreased, the water holding capacity (WHC) declined, while the water solubility index (WSI) and oil holding capacity (OHC) increased. In tumor cell proliferation inhibition assays, all three fractions inhibited the proliferation of MCF-7, HepG2, A549, and SGC-7901 cells in a concentration-dependent manner and maintained a high cell survival rate (>70%) in normal HUVECs; among them, SH-40 showed the lowest half-maximal inhibitory concentration (IC_50)_ value (1.09–3.89 mg/mL) (*p* < 0.05). SH-40 displayed the highest biological activity due to its higher molecular weight and characteristic monosaccharide ratio (*p* < 0.05), suggesting that molecular weight was one of the important factors influencing its function and activity. These findings provided a basis for the hierarchical preparation and differentiated application of polysaccharides isolated from *Sanghuangporus vaninii*.

## 1. Introduction

*Sanghuangporus vaninii* (*S. vaninii*), also known as *Phellinus vaninii*, belongs to the kingdom Basidiomycota, class Agaricomycetes, order Hymenochaetales, and family Hymenochaetaceae [1]. In China, *Sanghuang* had a long history of medicinal and edible use, and it was commonly used in folk practice by decocting its fruiting body in water to prepare daily health drinks [2,3]. In traditional Chinese medicine practice, this fungus was frequently employed to treat diarrhea, uterine bleeding, amenorrhea, and gastrointestinal dysfunction [4]. Its excellent water solubility made *S. vaninii* polysaccharides the primary components of its decoction, and modern pharmacological studies confirmed that these polysaccharides were precisely the key active substances responsible for the various biological activities observed in *Sanghuang* water extracts, such as antitumor, antioxidant, and hepatoprotective effects [5,6,7]. Previous research showed that different fungal polysaccharides exhibited distinct biological activities due to variations in their molecular weights. Dai et al. [8] obtained six polysaccharide fractions from *Cordyceps militaris* by fractional alcohol precipitation, and found that the low-molecular-weight fraction LMW-CMP (3.06 × 10^3^ Da) showed the strongest inhibition of HepG2 cell proliferation via the MAPK/NF-*κ*B pathway. Hsiao et al. [9] isolated POP-40, POP-60, and POP-80 from *Pleurotus ostreatus* by gradient ethanol precipitation, and found that the high-molecular-weight fraction POP-80 (6.71 × 10^5^ Da) showed the best efficacy against UVA-induced photoaging in Hs68 cells. The aforementioned studies suggested that molecular weight might be one of the key structural factors influencing the activity of fungal polysaccharides. However, there were still few reports on the structure, function, and biological activity of *S. vaninii* polysaccharides with different molecular weights.

Common separation techniques available for isolating polysaccharide components with different molecular weights included column chromatography, membrane filtration, and gradient ethanol precipitation; among these methods, column chromatography and membrane filtration employed specific molecular weight cut off values, but their high costs and prolonged processing times limited their application in food industry production [8,10]. In contrast, the gradient ethanol precipitation (GEP) method leveraged the solubility differences in polysaccharides at varying ethanol concentrations to achieve stepwise separation based on molecular weight; this method was straightforward to operate, low-cost, employed mild conditions, and was easy to scale up [11], and was successfully applied to the isolation of polysaccharides from various fungi, such as *Cordyceps militaris* [8], *Pleurotus ostreatus* [9], and *Pholiota nameko* [12]. Therefore, utilizing GEP to obtain polysaccharide components with different molecular weights from *Sanghuang* represented an economical and efficient separation approach; conducting a systematic comparison of their function and biological activity differences using this method held significant importance for its precise application in the research and development of functional foods or pharmaceuticals.

Cancer has become the second leading cause of death worldwide, severely impairing patients’ quality of life and imposing a substantial socioeconomic burden [13]. Traditional anticancer drugs often exhibited toxic side effects and were not suitable for long-term use [14]; consequently, the search for highly safe auxiliary bioactive compounds suitable for long-term dietary interventions emerged as a key research direction in the field of food science. Edible fungal polysaccharides, owing to their favorable biocompatibility, and diverse pharmacological activities, particularly their low toxicity and oral administration capability, showed unique advantages in the development of functional foods for cancer adjunct therapy [15]. The antitumor activity of *S. vaninii* polysaccharides was reported in various cell lines, including those representing hepatocellular carcinoma, lung cancer, breast cancer, and gastric cancer [16,17], but most existing studies focused on crude extracts or specific fractionated components. There remained a significant lack of cross-comparative studies investigating the inhibitory effects of *S. vaninii* polysaccharide components with different molecular weights against diverse types of tumor cells.

Building upon the aforementioned research landscape, this study focused on the fruiting body polysaccharides of *S. vaninii*, employing a gradient ethanol precipitation method (40%, 60%, 80%) to perform a simplified fractionation of the crude polysaccharide mixture, thereby obtaining polysaccharide fractions with distinct molecular weight ranges. Through systematic characterization of these fractions combined with an in vitro inhibition of tumor cell proliferation screening using tumor cell lines such as HepG2 and MCF-7, this study aimed to elucidate the differences in the physicochemical properties and inhibition of tumor cell proliferation among the various polysaccharide fractions obtained via the gradient alcohol precipitation process, thereby providing a scientific basis for the precise fractionation and preparation of *S. vaninii* polysaccharides and their differentiated applications in functional foods and pharmaceuticals.

## 2. Materials and Methods

### 2.1. Materials and Reagents

The dried fruiting bodies of *S. vaninii* were purchased from Bajiazi Town, Helong City, Jilin Province, China. A series of monosaccharide standards, including glucosamine (GlcN), mannose (Man), ribose (Rib), glucose (Glc), xylose (Xyl), arabinose (Ara), rhamnose (Rha), galacturonic acid (GalA), galactose (Gal), glucuronic acid (GlcA), and fucose (Fuc), were purchased from Shanghai Aladdin Bio-Chem Technology Co., Ltd. (Beijing, China), while dextran (national drug reference material) was obtained from the National Institutes for Food and Drug Control (NIFDC). Human breast cancer (MCF-7) cell lines, human hepatocellular carcinoma (HepG2) cell lines, human gastric adenocarcinoma (SGC-7901) cell lines, human non-small cell lung cancer (A549) cell lines and human umbilical vein endothelial (HUVEC) cell lines were obtained from the Cell Culture Center, Institute of Basic Medical Sciences, Chinese Academy of Medical Sciences. The other chemical reagents used were analytical grade or high-performance liquid chromatography (HPLC) grade.

### 2.2. Preparation of Polysaccharides

The crushed fruiting bodies of *S. vaninii* (4 kg) were subjected to hot-water extraction three times (90 °C, 2 h each). A total of 40 L of water was used, divided into three approximately equal portions (approximately 13.3 L per extraction). The extracts were combined. After the extract reached room temperature, it was centrifuged at 6000 rpm for 20 min to remove residual solids. All supernatants were combined and concentrated under reduced pressure at 50 °C and −0.09 MPa until the volume reached approximately 4 L. Concentration was stopped once the desired volume was achieved. The concentrate was adjusted with ethanol to a final concentration of 40% (*v*/*v*) and then kept overnight at 4 °C to permit polysaccharide precipitation. The mixture was centrifuged, and the precipitate was separated from the supernatant; the 40% ethanol precipitate was collected and designated as Crude SH-40. The remaining supernatant was adjusted with ethanol to a final concentration of 60% (*v*/*v*). After standing overnight at 4 °C, the mixture was centrifuged, and the resulting precipitate was collected and named Crude SH-60. The remaining supernatant was then adjusted with ethanol to a final concentration of 80% (*v*/*v*). After standing overnight at 4 °C, the mixture was centrifuged, and the resulting precipitate was collected and named Crude SH-80. The three aforementioned precipitates were collected and each was dissolved in an appropriate volume of deionized water to a final polysaccharide concentration of approximately 5 mg/mL. The Sevag reagent was prepared by mixing chloroform and n-butanol at 4:1 (*v*/*v*). This reagent was then added to the polysaccharide solution at a ratio of 4:1 (*v*/*v*), and the mixture was vigorously shaken in a fume hood for 30 min. After centrifugation at 4 °C and 4000–5000 rpm for 30 min, the supernatant was collected and lyophilized, yielding SH-40, SH-60, and SH-80.

### 2.3. Determination of Fourier Transform Infrared (FT-IR) Spectroscopy and Specific Optical Rotation

To determine the functional groups and structural properties of SH-40, SH-60, and SH-80, FT-IR spectroscopy was employed. Each polysaccharide sample (2 mg) was mixed with anhydrous KBr (100 mg) and compressed into a transparent pellet. Spectral acquisition was performed using an FT-IR spectrometer (Nicolet iS20, Thermo Scientific, Waltham, MA, USA) across the 4000–400 cm^−1^ region. Background noise was corrected by reference to the KBr blank. Subsequently, the specific optical rotation was measured. Each sample (8 mg) was dissolved in distilled water (8 mL) to prepare a 1 mg/mL solution, with distilled water as the blank. The specific rotation ([*α*]D^20^) of the three polysaccharides was measured using an optical rotation meter (Autopol I, Rudolph Research Analytical, Hackettstown, NJ, USA) at a wavelength of 589 nm and a temperature of 20 °C.

### 2.4. Determination of Molecular Weight (MW) and Homogeneity

The molecular weight characterization of SH-40, SH-60, and SH-80 was determined using a high-performance gel permeation chromatography (HPGPC) system (BOCL104, Shimadzu, Kyoto Prefecture, Japan) fitted with a Shodex SUGAR KS-804 (Resonac Corporation, Tokyo, Japan) column (8.0 mm × 300 mm) and an RID-20A refractive index detector. After dissolution in ultrapure water at 1 mg/mL, each sample was passed through a 0.22 µm membrane and then injected for analysis [18]. Ultrapure water was used as the mobile phase at a flow rate of 1.0 mL/min; 50 μL was injected, and measurements were performed at a constant 40 °C. The LabSolutions workstation was used for data acquisition and processing. Molecular weight calibration was carried out using dextran standards of known molecular weights under the same chromatographic conditions; a calibration curve was then established by plotting retention time against log Mw. From their retention times, the relative molecular weights of the samples were calculated by means of the calibration curve.

### 2.5. Determination of Monosaccharide Composition

The determination of monosaccharide composition was performed using a high-performance liquid chromatography (HPLC) system (1260 Infinity II, Agilent, Santa Clara, CA, USA) associated with the 1-phenyl-3-methyl-5-pyrazolidone (PMP) pre-column derivatization method. HPLC was performed using a 1260 VWD detector and an Agilent 5 TC-C18 chromatographic column (4.6 mm × 250 mm, 5 μm). The methodology was referenced from previous reports with minor modifications [19]. Each sample (10 mg) was treated with TFA (2 mol/L, 3 mL) solution in a reaction vessel and hydrolyzed at 120 °C for 6 h in a constant-temperature oven. After transfer to a rotary evaporator, the hydrolysate was evaporated to dryness with anhydrous methanol to eliminate residual TFA completely. The dried sample was taken up in water, and PMP-methanol solution (0.5 mol/L, 200 μL) and NaOH solution (0.3 mol/L, 200 μL) were introduced sequentially. The derivatization was allowed to proceed in a 70 °C water bath for 1 h, followed by quenching with hydrochloric acid. The obtained product was subjected to repeated chloroform partitioning, after which the aqueous layer was recovered. This aqueous fraction was then analyzed by HPLC. The chromatographic separation employed a mobile phase composed of 17% acetonitrile and 83% phosphate buffer, with UV detection at 245 nm, a column oven maintained at 35 °C, an elution rate of 0.8 mL/min, and a sample injection volume of 20 μL. For monosaccharide composition analysis, eleven monosaccharide standards were used as calibration references to construct a standard curve, from which the molar percentages of the monosaccharides were calculated.

### 2.6. Methylation Analysis

To characterize the glycosidic linkages, methylation analysis was conducted on each sample according to reference [20] with minor modifications. To eliminate interference from uronic acid residues during methylation, reduction of these residues was required. A 20 mL portion of CSP-A solution (1.0 mg/mL) was mixed with carbodiimide (500 mg), and the resulting mixture was agitated at pH 4.75 for 3 h. Subsequently, a sodium borodeuteride (NaBD_4_) solution (15 mL, 5 mg/mL) was introduced to reduce the uronic acids present in the polysaccharide. The pH was then brought to 7.0 using 2 M HCl, and agitation was continued for another 2 h. This reduction cycle was carried out twice. The reduced polysaccharide obtained was then pre-column derivatized with PMP, and the amounts of glucuronic acid and glucose in the reduced product were quantified. For each reduced polysaccharide sample, dissolution was first performed in DMSO (4 mL) at ambient temperature (18–40 °C), after which sodium hydroxide (300 mg) dissolved in DMSO (2 mL) was added. After incubation for 30 min with shaking, during which the yellowish flocculent material fully dissolved, the mixture was transferred to an ice bath for cooling. After ice cooling, the mixture was treated with 2 mL of CH_3_I protected from light and subsequently ultrasonicated for 45 min. This treatment cycle was performed on three separate occasions. The derivatization was stopped by adding 2 mL of distilled water, and the resulting solution was concentrated under reduced pressure until gas evolution ceased. The final product was dialyzed through a 3500 Da cut off cellulose membrane over a period of 48 h. The dialyzed, dried product was subjected to hydrolysis in TFA (6 mL, 2 mol/L) at 110 °C for 3 h. After the TFA was removed, NaBH_4_ (10 mg/mL) was added, and the reduction reaction was conducted under vigorous agitation for 3 h. The reaction was terminated with acetic acid, the pH was adjusted to 5.5–7.0, followed by three rounds of co-evaporation with methanol, and finally acetylated at 100 °C for 2 h. Chloroform extraction was performed three times; the pooled chloroform phases were dried thoroughly and then analyzed by gas chromatography-mass spectrometry (GC-MS) using an Agilent system (8860 5977b, Agilent, Santa Clara, CA, USA). The sample was maintained at 140 °C for 2 min, then heated at a rate of 2 °C/min to 200 °C and held for 2 min, followed by further heating at 10 °C/min to 280 °C; helium gas was used as the carrier gas with a constant flow rate of 1 mL/min. The linkage patterns of the polysaccharide residues were identified by comparing the obtained mass spectra with a public database (https://glygen.ccrc.uga.edu/ccrc/specdb/ms/pmaa/pframe.html (accessed on 30 June 2026)), and the molar ratios of each linkage type were determined based on the integral areas of the corresponding chromatographic peaks.

### 2.7. Congo Red Assay

The Congo red method was employed to preliminarily assess whether the polysaccharides from SH might adopt a triple-helix conformation; the specific procedure followed the method described in reference [21] with minor modifications. Congo red solution (80 μmol/L, 2.0 mL) was mixed separately with three sample solutions (1.25 mg/mL, 2.0 mL each) and a blank aqueous solution. Subsequently, NaOH solution (1 mol/L) was gradually introduced to reach final NaOH concentrations of 0, 0.2, 0.4, 0.6, 0.8, and 1.0 mol/L. Incubation was carried out at room temperature for 5 min. Finally, spectra were recorded in the range of 200–800 nm using an ultraviolet–visible (UV–Vis) spectrophotometer (UV2600, Techcomp, Shanghai, China), and the wavelength corresponding to the maximum absorption peak was measured.

### 2.8. Thermal Gravimetric (TG) Analysis

The polysaccharide content was determined using a TG analyzer (TG209F1 Libra, NETZSCH, Selb, Germany). A suitable amount of each sample was put into a ceramic crucible, and the system was purged with nitrogen at 20 mL/min, which also served as the carrier gas. Heating was then carried out from 30 to 800 °C at 20 °C/min. Mass changes were continuously recorded as a function of temperature, and the residual percentage and polysaccharide content were determined by analyzing the weight loss steps on the TG curve. The nitrogen flow rate was maintained constant throughout the heating process to ensure efficient removal of pyrolysis products and to prevent secondary reactions.

### 2.9. Scanning Electron Microscopy (SEM) Analysis

The surface morphology of the polysaccharides was observed by SEM (Gemini 360, ZEISS, Oberkochen, Germany). The three samples were dried, gold-coated, and mounted on a copper stage. Imaging was performed at selected acceleration voltages with magnifications of 200×, 1000×,1500×, and 2000× respectively. High-magnification SEM micrographs revealed the microstructural characteristics of the samples, including particle shape, surface texture, and agglomeration behavior.

### 2.10. Determination of Water Solubility Index (WSI)

The dried sample (M_0_, 30 mg) was accurately weighed, transferred into a centrifuge tube, and then dispersed in 1 mL of distilled water by vortexing at 5 s^−1^ for 30 min for WSI determination. After centrifugation at 3000 rpm for 30 min to pellet the insoluble residues, the supernatant was carefully removed. The precipitate was then dried at 50 °C until a constant weight was reached and weighed as M. WSI was determined as follows:

(1) $$Water\;Solubility\;Index(g/g)\;=\frac{M_{0}-M}{M_{0}}$$

### 2.11. Determination of Water Holding Capacity (WHC) and Oil Holding Capacity (OHC)

For WHC and OHC determination, an accurately weighed dried sample (M_1_, 100 mg) was transferred to a centrifuge tube, and 10 mL of distilled water or edible oil was then added. Vortexing was performed thoroughly, and the mixture was then held at room temperature for 30 min to permit complete adsorption. The mixture was then spun at 3000 rpm for 30 min to discard the unbound liquid. The supernatant was carefully discarded, and the centrifuge tube was inverted for 10 min to drain residual liquid. The remaining sample mass was recorded as M_2_. WHC and OHC were calculated using the following formulas:

(2) $$Water\;Holding\;Capacity(g/g)=\frac{M_{2}-M_{1}}{M_{1}}$$

(3) $$Oil\;Holding\;Capacity(g/g)=\frac{M_{2}-M_{1}}{M_{1}}$$

### 2.12. Cell Viability Assay

The culture conditions were slightly modified with reference to the literature [22]. Five cell lines (MCF-7, HepG2, A549, SGC-7901, and HUVEC) were passaged in DMEM medium containing 10% (*v*/*v*) FBS at 37 °C under 5% CO_2_. Log-phase cells were plated in 96-well plates (5 × 10^3^ cells/well) and allowed to adhere for 24 h, after which they were treated with SH-40, SH-60, or SH-80 (0.05, 0.1, 0.5, 1.0, or 2.0 mg/mL) for 48 h. Cell viability was assessed using the CCK-8 assay: each well received CCK-8 solution and was incubated for a further 2 h; absorbance values were determined at 450 nm using a Varioskan Flash full-wavelength scanning reader (Thermo Fisher Scientific, Waltham, MA, USA). Cells treated with the test substance were cultured in complete medium throughout the experiment as negative controls. SH polysaccharides were evaluated for inhibition of cellular proliferation by CCK-8 assay with blank and solvent controls. HUVECs were used to assess normal cell cytotoxicity. IC_50_ and 95% CI were calculated using a three-parameter logistic model in GraphPad Prism.

(4) $$\mathrm{Cell}\;\mathrm{viability}\;(\%)=\frac{{\mathrm{OD}}_{\mathrm{test}}\;-\;{\mathrm{OD}}_{\mathrm{blank}}}{{\mathrm{OD}}_{\mathrm{control}}\;-\;{\mathrm{OD}}_{\mathrm{blank}}}\;\times \;100\%$$

(5) $$\mathrm{Inhibition}\;\mathrm{rate}\;(\%)=\left[1\;-\;\frac{{\mathrm{OD}}_{\mathrm{test}}\;-\;{\mathrm{OD}}_{\mathrm{blank}}}{{\mathrm{OD}}_{\mathrm{control}}\;-\;{\mathrm{OD}}_{\mathrm{blank}}}\right]\;\times \;100\%$$

### 2.13. Statistical Analysis

Triplicate experiments were carried out, with data shown as mean ± SD. Statistical testing, including ANOVA and Pearson correlation analysis, was conducted in SPSS 17.0 and GraphPad Prism 6.0; statistical significance was set at *p* < 0.05.

## 3. Results and Discussions

### 3.1. Physical and Chemical Property Determination

The yield and chemical composition of *S. vaninii* polysaccharides were presented in Table 1. An increase in ethanol concentration from 40% to 80% resulted in a rise in crude polysaccharide yield (3.59.2–10.21%) and total sugar content (38.7–45.4%), while protein content also increased accordingly (2.9–4.3%). This indicated that the gradient ethanol precipitation method not only enabled the separation of polysaccharide fractions with different molecular weights but also resulted in fractions exhibiting systematic variations in their chemical composition: the fractions precipitated at higher ethanol concentrations exhibited higher polysaccharide purity and lower protein impurity levels. Furthermore, the purified SH-40, SH-60, and SH-80 all had a total sugar content greater than 82%. Compared with the other two groups, SH-80 had significantly higher total sugar and uronic acid contents (*p* < 0.05). And no proteins were detected, indicating that they all have good purity.

### 3.2. Determination of FT-IR Spectroscopy and Specific Optical Rotation

The functional groups of three *S. vaninii* polysaccharides (SH-40, SH-60, and SH-80) were characterized by FT-IR spectroscopy. As illustrated in Figure 1, all samples had typical polysaccharide absorption bands at 3400 and 2920 cm^−1^, which were assigned to O-H and C-H stretching vibrations, respectively [23]. Absorption at 1620 cm^−1^ originated from C=O asymmetric stretching, while that at 1400 cm^−1^ corresponded to C-H bending vibration [24]. The intense absorption region between 1200 and 1000 cm^−1^ corresponded to coupled stretching vibrations of C-O-C, C-O-H, and O-C-O within the pyranose ring [8]. The presence of *β*- and *α*-glycosidic bonds was suggested by the absorption peaks at 890 and 805 cm^−1^, respectively [5]. SH-40, SH-60, and SH-80 showed essentially identical absorption peak positions, indicating the same primary functional groups, but certain spectral bands differed in intensity. Since these fractions were precipitated at different ethanol concentrations, ethanol concentration might have affected the precipitation of components differing in polarity, molecular weight, and aggregation state, thereby altering functional group content and intermolecular hydrogen bonding. These intensity differences might therefore reflect such variations among the fractions. Meanwhile, the specific optical rotation ([*α*]D^20^) values for SH-40, SH-60, and SH-80 were +3.03°, −2.40°, and −6.00°, respectively. As the ethanol concentration increased, the specific optical rotation gradually changed from positive to negative, and the magnitude of the negative value increased. Overall, the SH-40, SH-60, and SH-80 compounds prepared using the GEP all exhibit similar functional groups.

### 3.3. Determination of Molecular Weight (MW) and Homogeneity

Using a calibration curve (log Mw = −0.4384x + 10.89), the weight-average molecular weights (Mws) of the three fractions (SH-40, SH-60, and SH-80) were determined by HPGPC. Figure 2 and Table 2 consistently showed that the single symmetric chromatographic peak and a polydispersity index ranging from 1.1 to 1.2 indicated the homogeneity of this polysaccharide. The molecular weights of SH-40, SH-60, and SH-80 were 5.90 × 10^5^ Da, 4.82 × 10^5^ Da, and 4.13 × 10^5^ Da, respectively. It is noteworthy that an increase in ethanol concentration results in prolonged retention times of polysaccharides within the chromatographic column, a trend that indirectly reflects a reduction in their molecular weight. This phenomenon was consistent with the trends observed by Dai et al. [8], Hsiao et al. [9], and Zhang et al. [25] when using the GEP method to precipitate polysaccharides and determine their molecular weights. It is hypothesized that this outcome was attributed to the decrease in polysaccharide molecular weight with increasing solubility, which in turn rises with elevated ethanol concentration [26]. The polarity of the mixed solvent was reduced by an increased ethanol concentration; the hydration layer of polysaccharides was disrupted, and their solubility decreased. As a result, high-molecular-weight polysaccharides precipitated preferentially at lower ethanol concentrations, while low-molecular-weight polysaccharides, owing to their higher solubility, required a higher ethanol concentration to precipitate. The GEP method had been proven capable of precipitating polysaccharide components with decreasing molecular weights [27]. The retention times of SH-40, SH-60, and SH-80 were similar to their molecular weights, which might be attributed to the unique conformation of SH groups resulting in minimal differences in solubility among these components at high alcohol concentrations.

### 3.4. Determination of Monosaccharide Composition

Figure 3 shows the HPLC chromatograms of standard monosaccharides and three SH polysaccharides, respectively. Four neutral sugars (Man, Rha, Glc, Gal), two uronic acids (GlcA, GalA), and one amino sugar (GlcN) were detected in SH-40, SH-60, and SH-80. Table 3 presents the monosaccharide molar percentages of these polysaccharides. Although the monosaccharide compositions of SH-40, SH-60, and SH-80 were predominantly composed of Man, Glc, and Gal, their molar ratios exhibited differences. All three polysaccharides were distinct heteropolysaccharides, with Glc showing the highest content and Rha the lowest. It is worth noting that, in the SH polysaccharides, the contents of Glc and GlcA consistently increased with rising ethanol concentration, whereas the levels of Gal and GalA exhibited an inverse trend. Similar selective alcohol precipitation phenomena have also been reported in studies of other fungal polysaccharides: Wang et al. [28] observed increased levels of Glc and GlcA from polysaccharides extracted from *Lentinus edodes* substrates as the alcohol precipitation concentration increased; and Hsiao et al. [9], in their study of *Pleurotus ostreatus* polysaccharides, identified the most pronounced variation in GalA content, indicating that alcohol precipitation concentration can alter the molar ratio distribution of monosaccharides. When the present results were considered together with these research findings, polysaccharides with high Glc content were found to readily precipitate at high ethanol concentrations; however, as the ethanol precipitation concentration decreased, the GalA content within these polysaccharides increased. This trend appeared to be widespread among various fungal polysaccharides.

These phenomena arose from structural differences in polysaccharides: glycans rich in Glc might adopt either a helical or extended conformation and contain fewer hydrophilic groups; under low-polarity conditions, they were prone to aggregation via hydrogen bonding and hydrophobic interactions. As the GalA content increased, the sugar chains might adopt an extended conformation and carry a high negative charge; they maintained solubility by forming stable hydrogen bonds with water molecules. Although GalA and GlcA both belong to the uronic acid class, GalA precipitated upon increasing ethanol concentrations due to the protonation of its carboxyl group and a consequent weakening of electrostatic repulsion. The latter, due to its unique conformation such as being embedded within the main chain or at the terminal end, was insensitive to changes in polarity. Overall, ethanol did not alter the types of monosaccharides; instead, it achieved fractionation solely through differences in intercomponent interactions.

### 3.5. Methylation Analysis

GC-MS was used to analyze the partially methylated alditol acetates (PMAAs) derived from the methylated polysaccharides, and a representative total ion current chromatogram was shown in Figure 4. The mass spectrometry data for the PMAAs were referenced against the Complex Carbohydrate Structure Database (CCSD) maintained by the Complex Carbohydrate Research Centre (CCRC) at the University of Georgia. The relative abundance of glycosidic bonds was summarized in Table 4. The methylation results indicated that the glycosidic bond compositions of the three polysaccharides were identical, with only differences in their relative abundances. Their glycosidic bond compositions primarily encompassed four residue types: Glc, Gal, Man, and GlcN. Among these, glucopyranosyl (Glc*p*) residues exhibited the most diverse linkage patterns, namely 1,6-Glc*p*, 1,2-Glc*p*, and 1,3-Glc*p*; mannopyranosyl (Man*p*) residues were present as 1,4-Man*p* and 1,3-Man*p*; galactopyranosyl (Gal*p*) residues were detected solely as 1,3-Gal*p*; additionally, glucopyranosamine (Glc*p*N) residues were identified as 1,3-Glc*p*N. GC-MS results indicated that the gradient ethanol precipitation method achieved fractionation primarily based on differences in molecular weight without altering the type of glycosidic bond.

### 3.6. Congo Red Assay

The Congo red method was commonly used to determine whether polysaccharides adopted a triple-helix conformation. As shown in Figure 5, for SH-60 and SH-80, the λmax after the Congo red reaction showed a pronounced red shift with increasing NaOH concentration, suggesting the formation of polysaccharide-Congo red complexes and the possible presence of a triple-helical conformation. As the NaOH concentration increased further, the λmax values remained stable. In contrast, the λmax values for SH-40 exhibited no characteristic red shift with variations in NaOH concentration compared to the control group. The literature reported that hydrogen bonds were the primary force maintaining polysaccharide triple helices: helical structures persist under low NaOH concentrations with distinct red shifts, while excessive NaOH concentration disrupts hydrogen bonding, leading to helix unwinding and diminished or absent red shifts [27]. Notably, even at sodium hydroxide concentrations as high as 1.0 mol/L, both SH-60 and SH-80 exhibited a stable red-shift phenomenon, indicating that the complexes formed with Congo Red were stable, enabling them to resist conformational unwinding in alkaline environments [29]. Future studies could employ circular dichroism and atomic force microscopy to further investigate their spatial conformation.

### 3.7. Thermal Gravimetric (TG) Analysis

Thermogravimetric (TG) analysis and differential thermogravimetric analysis (DTG) were employed to evaluate the thermal stability of the three polysaccharides. As shown in Figure 6, the TG curves of SH-40 and SH-60 exhibited two-stage weight loss, whereas SH-80 exhibited three-stage weight loss, indicating a more complex thermal degradation process for SH-80, in which further thermal decomposition of residual carbonaceous matter may occur during the high-temperature stage [30]. The first stage (30–199.1 °C) involved a weight loss ranging from 10.03% to 10.90%, primarily attributed to the evaporation of free water and bound water [31]. The third stage of the SH-80 process (269.1–799.3 °C) involved the further thermal decomposition of residual carbonaceous matter [30], with a weight loss rate of 48.26%. The second stage for SH-40 and SH-60 (199.1–799.3 °C) and for SH-80 (190.6–269.1 °C) represented the main degradation phase, with a weight loss ranging from 13.44% to 63.57%, which may be associated with glycosidic bond cleavage [32]; the most prominent DTG peak temperatures for these samples were 318.4 °C (SH-40), 322.2 °C (SH-60), and 316.0 °C (SH-80), respectively, indicating that SH-60 possessed the strong thermal resistance of its backbone. SH-80 exhibited the lowest degradation temperature. The residual mass ratio at 799.3 °C was highest for SH-40, followed by SH-80, and lowest for SH-60. SH-40 began its degradation earlier; its degradation rate was relatively gentle, with a broad weight loss peak. The SH-60 formulation exhibited concentrated and vigorous degradation, and it produced the lowest residual mass ratio. SH-80 degradation was highly abrupt, and its residual mass ratio fell between those of the two polysaccharides. Based on previous analytical results, the differences in thermal stability among these three polysaccharides could be systematically explained. SH-60 and SH-80 might possess helical structures; these properties enabled them to effectively resist thermal decomposition [33]. SH-40 contained a lower Glc content than SH-60 and SH-80, but the GalA content was higher than that of SH-60 and SH-80, and it exhibited the lowest thermal degradation temperature, which was similarly consistent with findings reported in the literature [34].

### 3.8. Scanning Electron Microscopy (SEM) Analysis

The surface morphology of SH-40, SH-60, and SH-80 was observed by scanning electron microscopy (SEM) at 200×, 1000×, 2000×, and 5000× magnifications. Figure 7 results indicated that under low magnification, SH-40 appeared as irregular bulk aggregates with a dense structure; under high magnification, its surface exhibited numerous pores, yet its overall morphology remained intact. This tightly packed morphology was typically associated with high molecular weight [35]. In contrast, SH-60 exhibited a loose, porous network-like structure with smaller particle sizes and surfaces covered by fine micropores; this structure might be linked to its intermediate molecular weight [36]. SH-80 was primarily composed of irregular bulk particles of varying sizes with poor dispersibility; its surface was rough and its particle size distribution was relatively concentrated, which was consistent with its low molecular weight [37].

### 3.9. Determination of Water Solubility Index (WSI), Water Holding Capacity (WHC), and Oil Holding Capacity (OHC)

The results of the determination of water solubility index (WSI), water holding capacity (WHC), and oil holding capacity (OHC) were shown in Table 5; as the alcohol precipitation concentration increased, the solubilities of SH-40, SH-60, and SH-80 were 0.86 ± 0.11, 0.94 ± 0.17, and 0.95 ± 0.13 g/g; their water holding capacities were 2.71 ± 0.20, 0.99 ± 0.26, and 0.56 ± 0.11 g/g; and their oil holding capacities were 3.29± 0.16, 3.63 ± 0.19, and 5.82 ± 0.21 g/g.

The WSI trend was closely associated with polysaccharide structure and molecular weight. Due to its relatively high molecular weight, SH-40 exhibited a dense bulk-like structure, making it difficult for water molecules to penetrate into its interior; consequently, its WSI was the lowest [38]. The SH-60 polymer possessed a moderate molecular weight; its loose, porous network structure increased the accessible contact area of the polysaccharide chain with water molecules, thereby facilitating the hydration process; consequently, its WSI value fell within an intermediate range. Due to its relatively low molecular weight, individual SH-80 molecules dispersed more readily in water; consequently, its WSI value was the highest.

WHC was not determined solely by molecular weight but was jointly regulated by solubility and aggregation state. SH-40 primarily consisted of insoluble aggregates, relying mainly on a physical network to retain water. However, the interaction between the retained water and the polymer was relatively weak, making it readily removable under centrifugal conditions. Consequently, its measured WHC was actually higher than those of SH-60 and SH-80. Although SH-60 and SH-80 exhibited strong hydrophilicity and high hydration capacity, their elevated WSI led to partial dissolution and loss of polysaccharides during testing, while their lower measured WHC was attributed to the absence of a three-dimensional framework structure formed through macromolecular chain entanglement. The present findings were in line with previous reports on polysaccharides from *Volvariella volvacea* [39] and isolated acorns [40]. The inverse relationship between the WSI and the WHC might arise from particle size, which was a key factor influencing the WSI, while the WHC depended on hydration sites or active sites, pore size, and conformational structure [40].

SH-80 exhibited the best OHC, which was primarily attributed to its lowest molecular weight. The low molecular weight promoted the formation of fine, fragmented particles during the drying process, increasing the specific surface area and physical adsorption sites. Simultaneously, the higher Glc content in this sample and the potential formation of a triple-helix conformation within its hydrophobic regions further enhanced its affinity and retention capacity for oils. In contrast, SH-40 and SH-60, due to their higher molecular weights, possessed more intact and dense particle structures with larger particle sizes, limited specific surface areas, and insufficiently exposed hydrophobic regions, resulting in OHC that were markedly lower than those of SH-80.

### 3.10. Cell Viability Assay

Blank controls were incorporated into all cell viability assays, and no significant differences were observed (*p* > 0.05). As shown in Figure 8, the inhibitory effects of the three polysaccharides on tumor cell viability were concentration-dependent. Over the 0.05–2 mg/mL range, SH-40 displayed the most potent cytotoxicity, especially against MCF-7 cells, for which inhibition was most marked (*p* < 0.05). At 2 mg/mL, SH-40 produced an inhibition rate of 73.53 ± 3.68% in MCF-7 cells, with a half-maximal inhibitory concentration (IC_50_) of 1.09 mg/mL. The IC_50_ values of SH-40 against HepG2, A549, and SGC-7901 cells were 1.51, 1.55, and 3.89 mg/mL, respectively. To assess potential cytotoxicity toward normal cells, experiments were performed with human umbilical vein endothelial cells (HUVECs). Across the same concentration range (0.05–2 mg/mL), SH polysaccharides showed relatively low toxicity to HUVECs, and cell viability remained above 70% in all cases. In the present study, the selectivity index was not derived from IC_50_ values; instead, normal-cell cytotoxicity was evaluated solely on the basis of cell viability. These results indicated that SH polysaccharide exerted only a weak inhibitory effect on HUVECs; nevertheless, its selectivity for tumor cells required further confirmation in additional experiments.

The inhibitory effect of SH polysaccharides on cell proliferation might be related to their structure-activity relationship [41]. Among the three components, SH-40 exhibited a relatively strong inhibitory effect, and its relatively high molecular weight suggested that molecular weight could be one of the factors influencing this activity. This finding was consistent with previous reports indicating that high-molecular-weight *Phellinus igniarius* mycelial polysaccharides exhibited stronger biological activity [42]. However, molecular weight was not the sole determining factor; in this study, the molecular weight, monosaccharide composition and molar ratio, glycosidic bond types, conformation, and physicochemical properties all changed simultaneously; therefore, the current results merely suggested that these factors might collectively influence cell proliferation, and a causal structure-activity relationship could not yet be established.

Based on the analysis of physicochemical properties, among the three components, SH-40 exhibited relatively higher molecular weight and WHC, while WSI and OHC were relatively lower; SH-60 and SH-80 showed the opposite trends. The magnitude of cell proliferation inhibition was generally consistent with the variation trends of molecular weight and WHC, but was inversely correlated with the variation trends of WSI and OHC. The higher WHC might be attributed to its higher molecular weight as well as interchain entanglement or aggregation, which could help maintain specific spatial conformations and thereby influence its interaction with cells; in contrast, the higher WSI and OHC primarily reflected the superior water solubility and lipid-binding capacity of the low-molecular-weight components, which predominantly influenced food processing behavior and were not directly equivalent to stronger cell proliferation inhibition activity.

Therefore, WSI, WHC, and OHC might influence the solubility and dispersion behavior of polysaccharides within food systems, yet they could not alone determine their inhibitory effect on cell proliferation. Molecular weight was one of the influencing factors, but it was not the sole determining factor. The aforementioned results only reflected the effects of these polysaccharides on cell proliferation and were insufficient to demonstrate their antitumor activity or selectivity. Future studies would further explore their structure-activity relationship and practical application potential by isolating homogeneous polysaccharides, elucidating their fine structural features, conducting mechanistic experiments, calculating a selectivity index based on IC_50_ values, and performing in vivo validation.

## 4. Conclusions

This study employed the gradient ethanol precipitation method (GEP) to isolate three polysaccharide fractions (SH-40, SH-60, and SH-80) from the fruiting bodies of *Sanghuangporus vaninii*. The results showed that the molecular weight of each fraction decreased in descending order as the ethanol precipitation concentration increased; although the monosaccharide composition and glycosidic linkage patterns were largely consistent among the fractions, differences were observed in their monosaccharide molar ratios, their functional activities as well as their in vitro inhibition of tumor cell proliferation efficacy which exhibited marked variations. These findings showed that the GEP method was not only an efficient crude fractionation technique but also a hierarchical purification approach capable of selective enrichment based on differences in functional activity, enabling the one-step isolation of specific polysaccharide fractions with distinct biological activities. From a process engineering perspective, a potential advantage of initiating precipitation at a lower ethanol concentration is that 40% ethanol preferentially precipitates the high-molecular-weight fraction (SH-40), which also exhibits the strongest inhibition of tumor cell proliferation. This may allow a highly active product to be obtained while reducing ethanol consumption and solvent recovery burden. However, this advantage is based only on laboratory-scale separation results; the feasibility and economic viability of this method for practical scale-up still require further validation through pilot- and industrial-scale experiments. Although this study indicated that SH polysaccharides exhibited potent inhibitory effects on tumor cell proliferation, their selectivity index, in vivo antitumor activity, and molecular mechanisms still require further investigation.

## Figures and Tables

**Figure 1:**
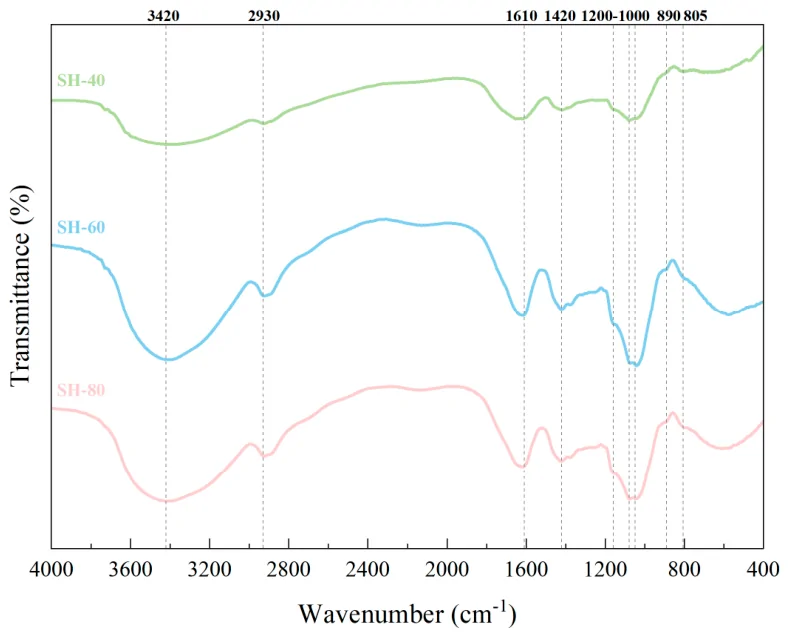
FT-IR spectra of SH-40, SH-60, and SH-80.

**Figure 2:**
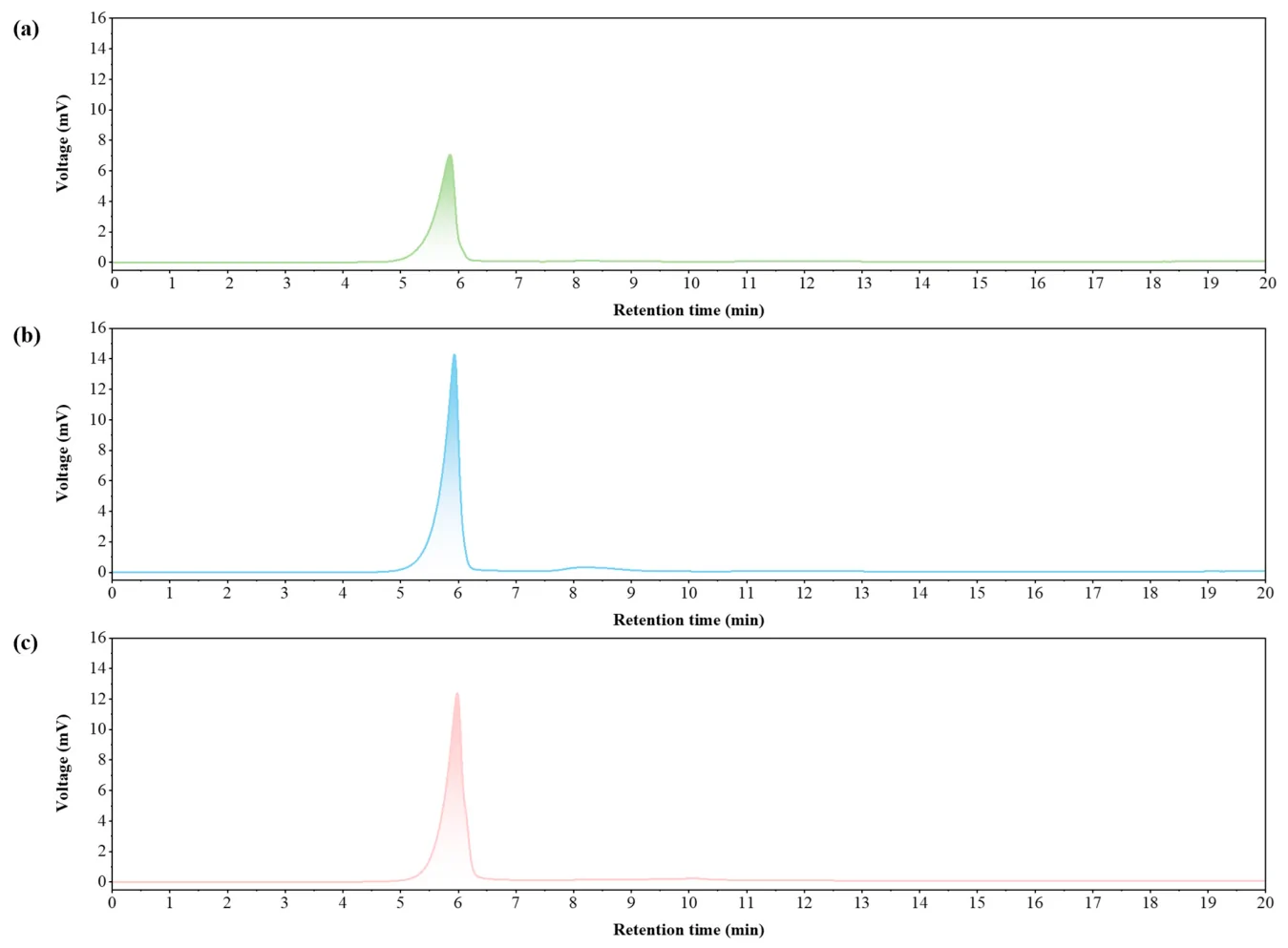
HPGPC chromatograms determining the molecular weights of SH-40 (**a**), SH-60 (**b**), and SH-80 (**c**).

**Figure 3:**
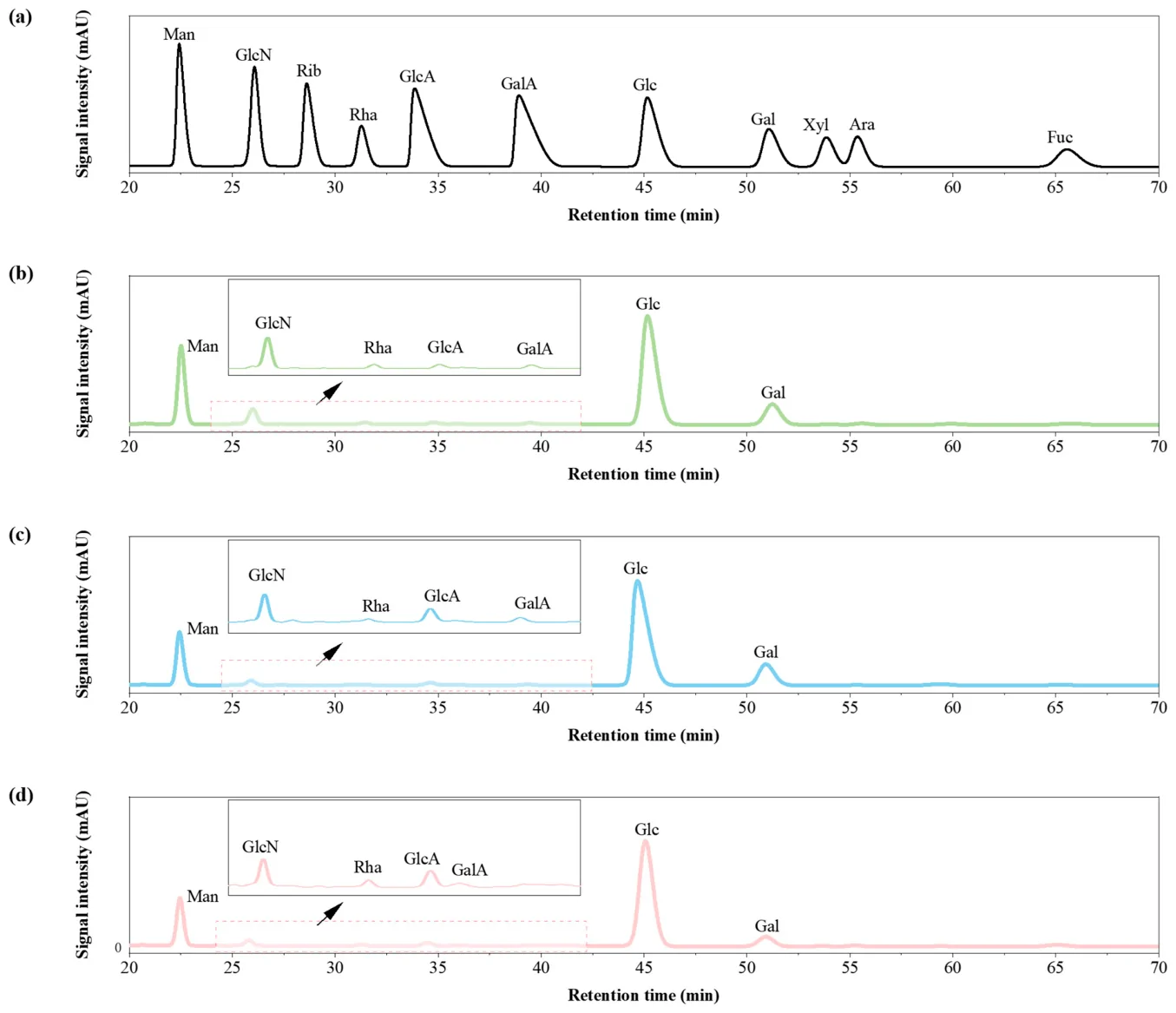
HPLC chromatograms determining the monosaccharide composition of standard samples (**a**), SH-40 (**b**), SH-60 (**c**), and SH-80 (**d**).

**Figure 4:**
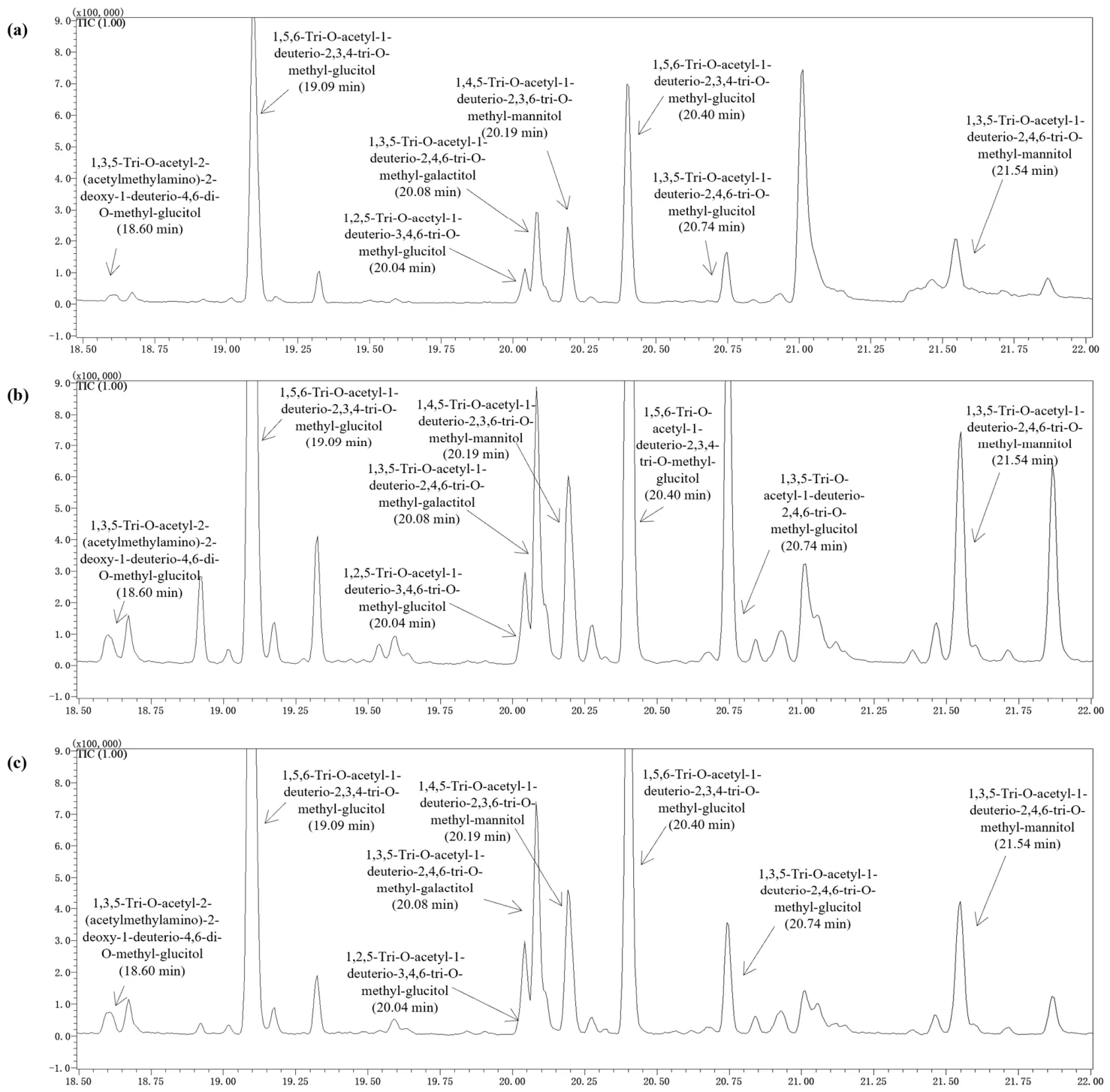
Total ion profile of partially methylated alditol acetates of SH-40 (**a**), SH-60 (**b**), and SH-80 (**c**).

**Figure 5:**
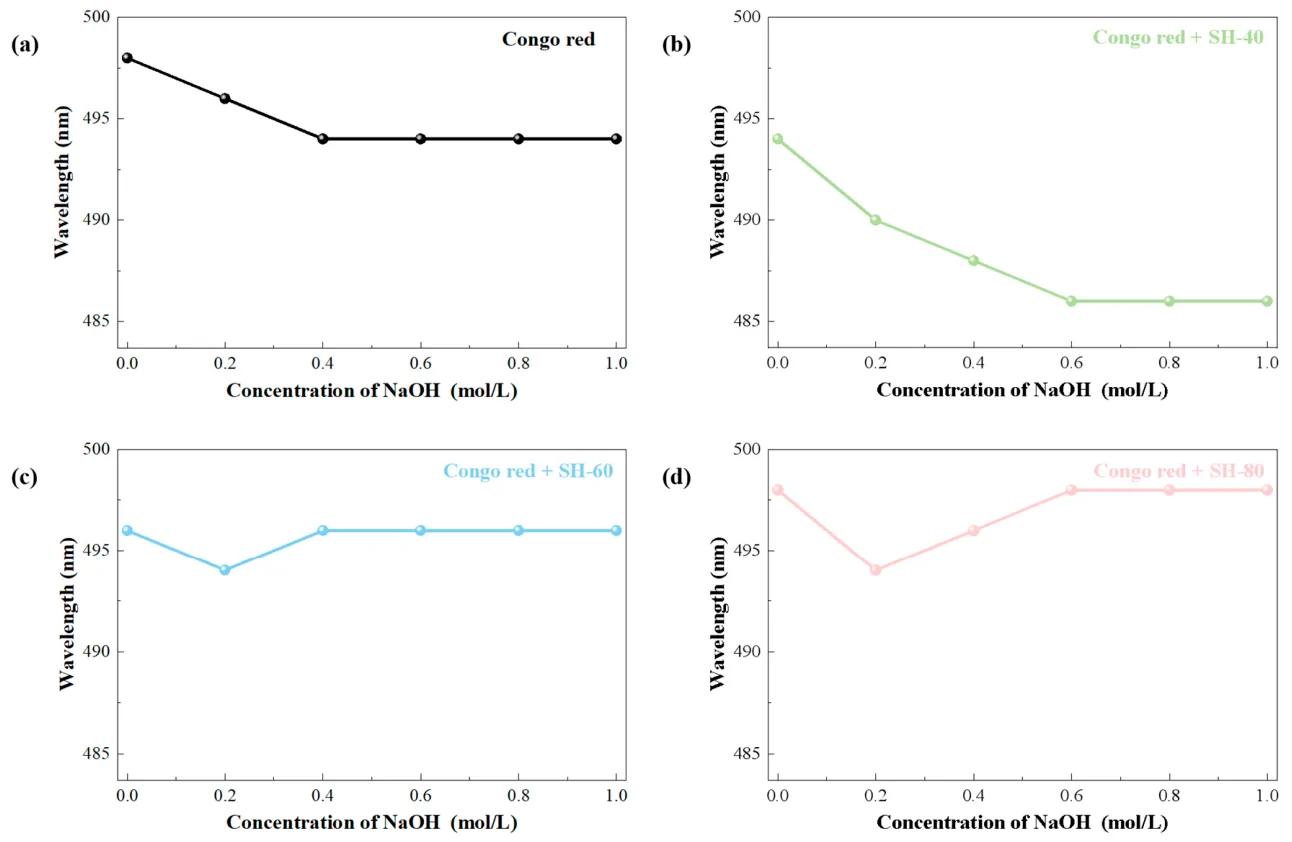
Maximum absorption wavelength changes in the Congo red (**a**), SH-40 (**b**), SH-60 (**c**), and SH-80 (**d**) mixture at various NaOH concentrations.

**Figure 6:**
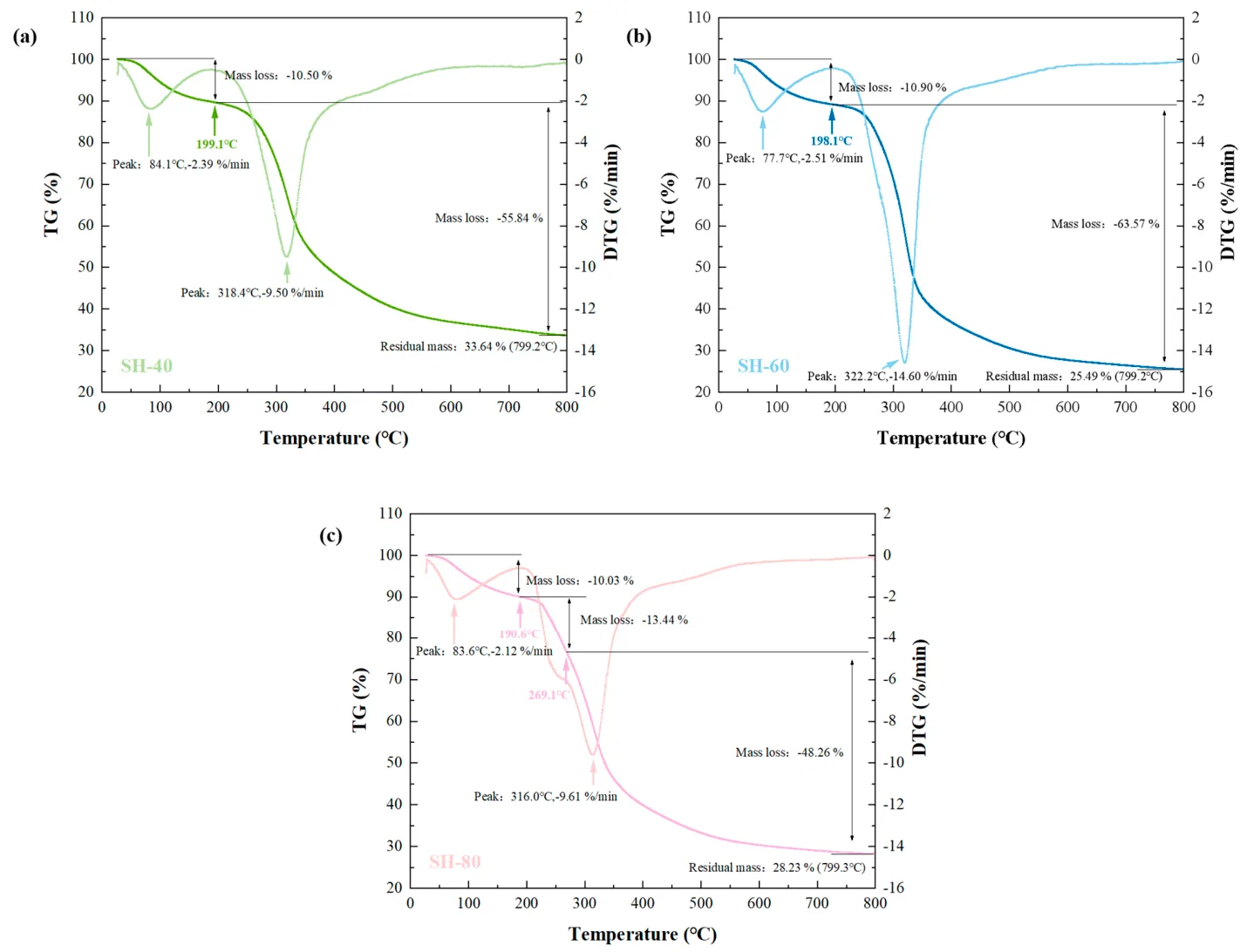
TG analysis of SH-40 (**a**), SH-60 (**b**), and SH-80 (**c**).

**Figure 7:**
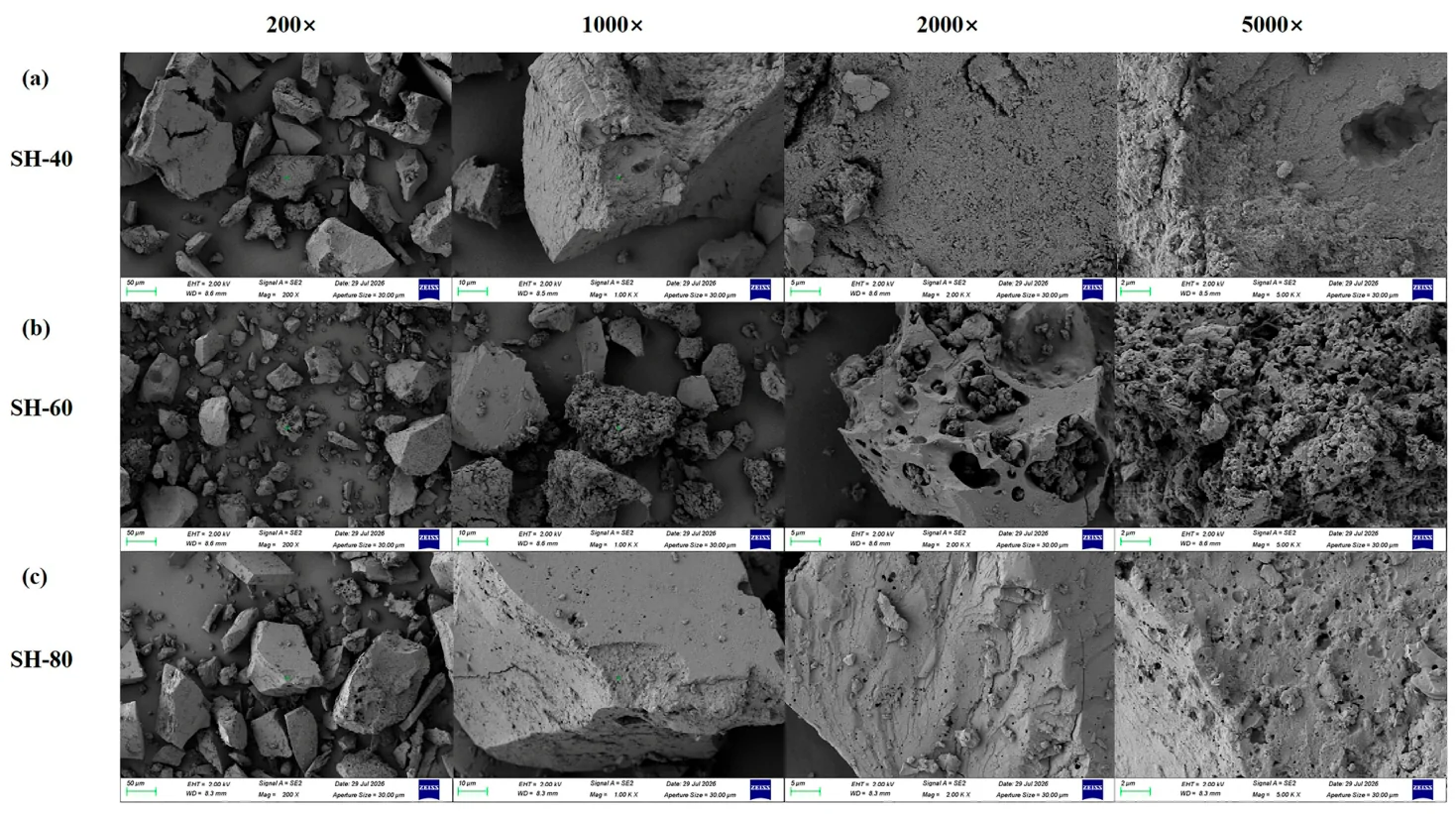
*SEM* analysis of SH-40 (**a**), SH-60 (**b**), and SH-80 (**c**).

**Figure 8:**
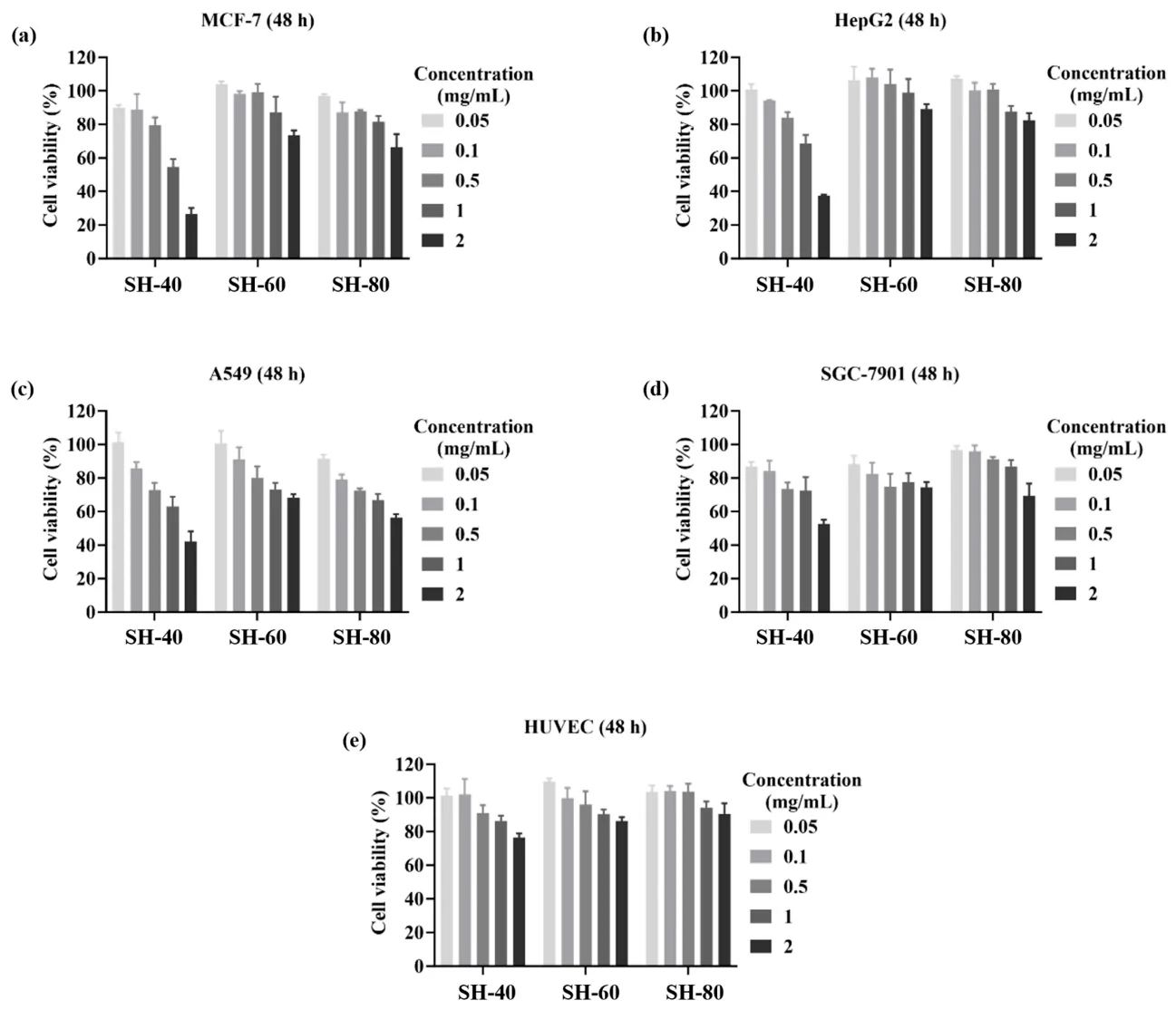
Effects of SH-40, SH-60 and SH-80 on cell viability of MCF-7 (**a**), HepG2 (**b**), A549 (**c**), SGC-7901 (**d**) and HUVEC (**e**) (*n* = 3).

**Table 1:** Comparison of the properties of SH polysaccharides.

	Crude SH-40	Crude SH-60	Crude SH-80
Yields (%)	3.59 ± 0.9	6.78 ± 0.7	10.21 ± 0.5
Total sugar content (%)	38.7 ± 0.8	40.1 ± 0.5	45.4 ± 0.6
Protein content (%)	2.9 ± 0.3	3.6 ± 0.2	4.3 ± 0.4
Uronic acid content (%)	0.9 ± 0.3	1.1 ± 0.2	1.7 ± 0.3
	SH-40	SH-60	SH-80
Total sugar content (%)	82.1 ± 2.3	84.2 ± 1.9	87.8 ± 2.1
Protein content (%)	-	-	-
Uronic acid content (%)	1.2 ± 0.6	1.3 ± 0.1	1.9 ± 0.5

Note: (*p* < 0.05); “-” indicated not detected.

**Table 2:** Molecular weight of SH polysaccharides.

	SH-40	SH-60	SH-80
**Molecular weight (Da)**	5.90 × 10^5^ Da	4.82 × 10^5^ Da	4.13 × 10^5^ Da
**Mw/Mn**	1.20	1.14	1.12

**Table 3:** Monosaccharide composition of SH polysaccharides.

Samples	Molar Percentage Ratio (%)						
	Man	GlcN	Rha	GlcA	GalA	Glc	Gal
**SH-40**	16.11	3.63	0.52	0.95	0.58	66.46	11.75
**SH-60**	11.55	1.17	0.20	1.00	0.26	72.80	13.02
**SH-80**	12.04	1.75	0.03	2.39	0.14	77.67	5.98

**Table 4:** The methylation analysis results of SH polysaccharides.

Retention Time (min)	PMAAs	Linkage Pattern	Molar Percentage Ratio (%)		
			SH-40	SH-60	SH-80
18.60	1,3,5-Tri-O-acetyl-2-(acetylmethylamino)-2-deoxy-1-deuterio-4,6-di-O-methyl-glucitol	1,3-Glc*p*N	1.18	1.22	1.42
19.09	1,5,6-Tri-O-acetyl-1-deuterio-2,3,4-tri-O-methyl-glucitol	1,6-Glc*p*	37.60	30.27	36.94
20.04	1,2,5-Tri-O-acetyl-1-deuterio-3,4,6-tri-O-methyl-glucitol	1,2-Glc*p*	3.79	2.84	4.23
20.08	1,3,5-Tri-O-acetyl-1-deuterio-2,4,6-tri-O-methyl-galactitol	1,3-Gal*p*	10.69	8.67	10.94
20.19	1,4,5-Tri-O-acetyl-1-deuterio-2,3,6-tri-O-methyl-mannitol	1,4-Man*p*	8.45	6.29	7.16
20.40	1,5,6-Tri-O-acetyl-1-deuterio-2,3,4-tri-O-methyl-glucitol	1,6-Glc*p*	24.58	29.51	26.24
20.74	1,3,5-Tri-O-acetyl-1-deuterio-2,4,6-tri-O-methyl-glucitol	1,3-Glc*p*	5.53	12.38	4.95
21.54	1,3,5-Tri-O-acetyl-1-deuterio-2,4,6-tri-O-methyl-mannitol	1,3-Man*p*	8.11	8.81	8.11

**Table 5:** WSI, WHC, and OHC of SH polysaccharides (*n* = 3).

	SH-40	SH-60	SH-80
WSI (g/g)	0.86 ± 0.11	0.94 ± 0.17	0.95 ± 0.13
WHC (g/g)	2.71 ± 0.20	0.99 ± 0.26	0.56 ± 0.11
OHC (g/g)	3.29± 0.16	3.63 ± 0.19	5.82 ± 0.21

Notes: Data were presented as means ± standard deviations (*p* < 0.05).

## Data Availability

The original contributions presented in this study are included in the article. Further inquiries can be directed to the corresponding authors.

## References

[1] Zhang H.N., Jiang F.C., Li L.Q., Liu X.Z., Yan J.K. (2022). Recent advances in the bioactive polysaccharides and other key components from *Phellinus* spp. and their pharmacological effects: A review. Int. J. Biol. Macromol..

[2] Wang H., Ma J.X., Zhou M., Si J., Cui B.K. (2022). Current advances and potential trends of the polysaccharides derived from medicinal mushrooms *sanghuang*. Front. Microbiol..

[3] Xue R.Y., Han W.Q., Gao X.Y. (2025). Inonotus hispidus has development potential in health and future foods. J. Future Foods.

[4] Wan X.L., Jin X., Xie M.L., Liu J., Andrey A.G., Wang H., Lv R.N., Liu D.Y., Wang Q., Li Y. (2020). Characterization of a polysaccharide from *Sanghuangporus vaninii* and its antitumor regulation via activation of the p53 signaling pathway in breast cancer MCF-7 cells. Int. J. Biol. Macromol..

[5] Li J.Y., Shi M.Q., Jia B.N., Liu S.L., Zhao Y. (2025). Structural characterisation of a polysaccharide from *Sanghuangporus vaninii* and its synergistic anti-hepatocellular carcinoma activity in combination with sorafenib. Int. J. Biol. Macromol..

[6] Li Z.H., Xu C.T., Pan J.L., Zhou Y., Li H.K., Li W.H., Zou Y.J. (2025). Augmentation of exopolysaccharide synthesis and its influence on biofunctional properties of polysaccharide in *Sanghuangporus vaninii via* targeted overexpression of phosphoglucomutase. Int. J. Biol. Macromol..

[7] Wei X.D., Qiao J.L., Wang J.J., Zhang M.X., Wang Z.F., Zeng Y.P. (2026). Phelligrin B, a flavonoid compound from *Sanghuangporus vaninii*, improves CCl_4_-induced acute liver injury associated with the PI3K-AKT1-mTOR pathway. Bioorg. Chem..

[8] Dai K.Y., Wang R.H., Mu L., Ji H.Y., Liu A.J. (2024). Structural characteristics and anti-tumor activity of alcohol-precipitated polysaccharides from *Cordyceps militaris* under different ethanol concentrations. Int. J. Biol. Macromol..

[9] Hsiao Y.F., Shao Y.C., Wu Y.T., Hsu W.K., Cheng K.C., Yu C.C., Chou C.H., Hsieh C.W. (2023). Physicochemical properties and protective effects on UVA-induced photoaging in Hs68 cells of *Pleurotus ostreatus* polysaccharides by fractional precipitation. Int. J. Biol. Macromol..

[10] Zhang X.R., Duan Y.Y., Xue J.F., Chen S., Wang H.Z. (2025). Edible mushroom polysaccharides: Structural characteristics, chemical modification strategies, and structure-activity relationship: A review. Int. J. Biol. Macromol..

[11] Li Z.X., Zhang X.Y., Zhu C.H. (2023). Physicochemical properties and Pb^2+^ adsorption capacity of freeze-dried hawthorn pectin fractions by gradient ethanol precipitation. Int. J. Biol. Macromol..

[12] Chou C.H., Sung T.J., Hu Y.N., Lu H.Y., Yang L.C., Cheng K.C., Lai P.S., Hsieh C.W. (2019). Chemical analysis, moisture-preserving, and antioxidant activities of polysaccharides from *Pholiota nameko* by fractional precipitation. Int. J. Biol. Macromol..

[13] Yang M.M., Mao S.Y., Duan J.A., Xiao P. (2026). The antitumor mechanism of polysaccharides through regulating the microbiota-metabolism-immune axis and their clinical application strategies. Carbohydr. Polym..

[14] Li C.Y., Zhou S.J., Chen S.Y., Xu Z., Xue Q.B., Wang H., Liu T., Chen W., Feng J., Chen J.J. (2025). Gut microbiota-mediated immunomodulation underlies the anti-tumor effects of a novel ginseng polysaccharide. Carbohydr. Polym..

[15] Liu Y., Yang L.Y., Wang J.H., Song L.Y. (2025). Physicochemical and colon cancer HT-29 cell inhibitory property of homogeneous polysaccharide from *Stropharia rugosoannulata*. Int. J. Biol. Macromol..

[16] Zhou X.Y., Zhang L., Hu W., Liu X.T., Song S.Y., Peng S.D., Wang D.J., Luo H., Wen Y., Qiao C.X. (2026). Characterization and antitumor activities of polysaccharides derived from *Inonotus sanghuang* against cancers of the liver and lungs. Int. J. Biol. Macromol..

[17] Liang H.Y., Ma Y.R., Zhao Y., Qayyum N., He F.T., Tian J.W., Sun X.Y., Li B., Wang Y.H., Wu M.Y. (2025). A Review on the Extraction, Structural Analysis, and Antitumor Mechanisms of *Sanghuangporus* Polysaccharides. Foods.

[18] Cai Y., Si Z.Y., Jiang Y., Ye M., Wang F., Yang X.B., Yu J.P., Gao X.D., Liu W. (2023). Structure-activity relationship of low molecular weight *Astragalus membranaceus* polysaccharides produced by *Bacteroides*. Carbohydr. Polym..

[19] Wang J.D.T., Xu X., Zou X.Q., Zhang R.F., Jia X.C., Dong L.H., Deng M., Zhang M.W., Huang F. (2024). Effect of ultrasound assisted H_2_O_2_ degradation on longan polysaccharide: Degradation kinetics, physicochemical properties and prebiotic activity. Int. J. Biol. Macromol..

[20] Wen C., Li T.T., Wang B.Q., Jin C., Li S.J., Li Y., Li M.X., Ding K. (2023). A pectic polysaccharide isolated from *Achyranthes bidentata* is metabolized by human gut *Bacteroides* spp. Int. J. Biol. Macromol..

[21] Yan X.H., Luo M., Zeng Z.L., Yang Y.J., Gao X.F., Gao Y., Cheng D., Zhang G.H., Gong D.M., Yu P. (2025). Physicochemical properties, prebiotic activities and application in yogurt of a novel polysaccharide from *Cinnamomum camphora* seed kernel. Future Foods.

[22] Xiao L.Y., Gong H.X., Tan X., Chen P., Yang Y.J., Zhu H., Zhong S.A. (2025). Physicochemical characterization and antitumor activity in vitro of a polysaccharide from *Christia vespertilionis*. Int. J. Biol. Macromol..

[23] Zhao C.F., Jin X.H., Li Z.G., Hu Y.F., Wei M.H., Chen Y.H., Wang R.Q., Song J.Y., Li Z.P., Zhang Y.F. (2026). *Sanghuangporus vaninii* polysaccharide attenuates chronic alcohol-induced liver injury based on modulation of AMPK/PPARα signaling pathway. J. Agric. Food Res..

[24] Qu Y.D., Yang H.X., Li S.Y., Li L.Z., Li Y., Wang D. (2023). The involvement of Th1 cell differentiation in the anti-tumor effect of purified polysaccharide from *Sanghuangporus vaninii* in colorectal cancer via multi-omics analysis. Int. J. Biol. Macromol..

[25] Zhang C., Hao L., Zhu Y.D., Zhang X.J., Zhao H.F., Zhang B.L. (2025). In vitro fermentation characteristics and modulation effects of polysaccharide fractions from *Schisandra sphenanthera* on intestinal microflora. Int. J. Biol. Macromol..

[26] Hu X.T., H. Douglas G. (2018). Fractionation of polysaccharides by gradient non-solvent precipitation: A review. Trends Food Sci. Technol..

[27] Yang X., Cao D.L., Ji H.Y., Xu H.J., Feng Y.Y., Liu A.J. (2023). Physicochemical characterization, rheological properties, and hypolipidemic and antioxidant activities of compound polysaccharides in Chinese herbal medicines by fractional precipitation. Int. J. Biol. Macromol..

[28] Wang K.J., Guo J.T., Cheng J.X., Zhao X.H., Ma B.H., Yang X.B., Shao H.J. (2021). Ultrasound-assisted extraction of polysaccharide from spent *Lentinus edodes* substrate: Process optimization, precipitation, structural characterization and antioxidant activity. Int. J. Biol. Macromol..

[29] Wang H.L., Chen Y.P., Zhang K., Lu J., Zhao X., Zhang Y.M. (2026). Two novel polysaccharides from *Spatholobus uniauritus*: Extract optimization, purification, structural characterization, hypoglycemic and immunomodulatory effects. Ind. Crops Prod..

[30] Ma Y.Q., Xiu W.Y., Wang X., Yu S.Y., Luo Y., Gu X.Y. (2022). Structural characterization and in vitro antioxidant and hypoglycemic activities of degraded polysaccharides from sweet corncob. J. Cereal Sci..

[31] Gao L.L., Song L., Ji J.H., Yang S.S., Wang F., Chen J.F., Liu D., Wang W., Han B.X. (2026). Structural characterization and antitumor activity of a polysaccharide from *Polygonatum cyrtonema* Hua. Int. J. Biol. Macromol..

[32] Hajji M., Hamdi M., Sellimi S., Ksouda G., Laouer H., Li S., Nasri M. (2019). Structural characterization, antioxidant and antibacterial activities of a novel polysaccharide from *Periploca laevigata* root barks. Carbohydr. Polym..

[33] Huang X.B., Zhang Y.L., Xie N., Cheng J.W., Wang Y.B., Yuan S.F., Li Q., Shi R., He L., Chen M. (2023). Molecular Characterization and Bioactivities of a Novel Polysaccharide from *Phyllostachys pracecox* Bamboo Shoot Residues. Foods.

[34] Jiang S., Xie H.F., Zuo Y.M., Sun J., Wu D.X., Shu X.L. (2024). Structural and functional properties of polysaccharides extracted from three *Dioscorea* species. Int. J. Biol. Macromol..

[35] Tang J.L., Qin X.L., Ritva R.C.V., Yang X.L., Deng Y.C., Hou C.S., Yang X.S. (2025). Physicochemical, functional and antioxidant properties of four polysaccharides sequentially extracted from jute (*Corchorus olitorius* L.) leaves. Int. J. Biol. Macromol..

[36] Meng Y., Yao M.Y., Xu X.J. (2026). Chain conformation characterization of polysaccharides: Current strategies, challenges, and emerging technologies. Glycosci. Ther..

[37] Liu M.Y., Liu J.W., Li G.Q., Zhang D.X., Qin D., Wang L.B., Xu Y.Q. (2024). Functional properties, structural characteristics, and anti-complementary activities of two degraded polysaccharides from strawberry fruits. Int. J. Biol. Macromol..

[38] Xu M., Qi M.Y., Goff H.D., Cui S.W. (2020). Polysaccharides from sunflower stalk pith: Chemical, structural and functional characterization. Food Hydrocoll..

[39] Wang J., Zhao C.Y., Li P., Wang L., Li S.N. (2023). Structural Characteristics and Multiple Bioactivities of *Volvariella volvacea* Polysaccharide Extracts: The Role of Extractive Solvents. Foods.

[40] Shokouh A., Mahmoud S.Z., Sabihe S.Z., Farzaneh A., Hariom Y. (2019). Effects of different drying methods on the physicochemical properties and antioxidant activities of isolated acorn polysaccharides. LWT-Food Sci. Technol..

[41] Yuan Q.X., Zhao L.Y., Li Z.H., Harqin C., Peng Y.F., Liu J.K. (2018). Physicochemical analysis, structural elucidation and bioactivities of a high-molecular-weight polysaccharide from *Phellinus igniarius* mycelia. Int. J. Biol. Macromol..

[42] Wang Z.C., Zheng Y., Lai Z.R., Hu X.L., Wang L., Wang X.Q., Li Z.T., Gao M.J., Yang Y.H., Wang Q. (2024). Effect of monosaccharide composition and proportion on the bioactivity of polysaccharides: A review. Int. J. Biol. Macromol..

